# Anca-positive vasculitis with full-house nephropathy, an unusual association: a case report and review of literature

**DOI:** 10.1590/2175-8239-JBN-2020-0134

**Published:** 2021-01-20

**Authors:** Carlos Mauricio Martínez Montalvo, Laura Catalina Gutierrez, Carolina Perez, Harrison Herrera Delgado, Paula Corinna Martinez Barrios

**Affiliations:** 1 Universidad del Rosario, Bogota, Colombia.; 2 Universidad Nuestra Señora Del Rosario, Bogota, Colombia.; 3 Universidad Surcolombiana, Neiva, Colombia.; 4 Universidad de Boyacá, Tunja, Colombia.

**Keywords:** Lupus Erythematosus, Systemic, Anti-Neutrophil Cytoplasmic Antibody-Associated Vasculitis, Nefropatias, Glomerulonephritis, Lúpus Eritematoso Sistêmico, Vasculite Associada a Anticorpo Anticitoplasma de Neutrófilos, Nefropatias, Glomerulonefrite

## Abstract

Rapidly progressive glomerulonephritis is a medical emergency, with mortality around 20%. It is characterized by crescent glomerulonephritis and progressive loss of kidney function, hematuria, and proteinuria. Its classification is given by immunofluorescence detection of antibodies against glomerular basement membrane (Anti-MBG), immunocomplexes, or pauci-immune pattern. Its etiology should be based on clinical findings, immunological profile, age, sex, and histopathological characteristics. We present a case of a 27-year-old woman with symptoms consistent with rapidly progressive glomerulonephritis and biopsy findings of a full-house kidney nephropathy, with an early fatal outcome. An association of low incidence, as it is a case with a full-house pattern, and an autoimmune profile for negative systemic lupus erythematosus makes this a rare case. ANCA-associated vasculitis with full-house kidney disease was diagnosed, an unusual condition with up to 3% presentation and few reports in the literature, highlighting the importance of its reporting and contribution to the literature.

## Resumo

A glomerulonefrite rapidamente progressiva é uma emergência médica, com mortalidade em torno de 20%. É caracterizada por glomerulonefrite com crescentes e perda progressiva da função renal, hematúria e proteinúria. Sua classificação é dada pela detecção na imunofluorescência de anticorpos anti-membrana basal glomerular (Anti-MBG), imunocomplexos, ou padrão pauci-imune. Sua etiologia deve ser baseada em resultados clínicos, perfil imunológico, idade, sexo e características histopatológicas. Apresentamos o caso de uma mulher de 27 anos de idade com sintomas consistentes com uma glomerulonefrite rapidamente progressiva e achados de biópsia de uma nefropatia com padrão full-house que evoluiu com desfecho fatal precoce. A associação de um padrão full-house, que possui uma baixa incidência, com um perfil autoimune para lúpus eritematoso sistêmico negativo torna este um caso raro. Foi diagnosticado vasculite associada ao ANCA com doença renal com padrão full-house. Por se tratar de uma condição incomum com até 3% de apresentação e poucos registros na literatura, destacamos a importância de seu relato e sua contribuição para a literatura.

## Introduction

Rapidly progressive glomerulonephritis (RPGN) is a medical emergency characterized by rapid loss (days to months) of kidney function. It occurs more frequently as a nephritic syndrome presenting hypertension, proteinuria in the non-nephrotic range, impaired renal function, and hematuria. The pathophysiology of this condition is based on immunopathological processes that are classified into 3 types: glomerulonephritis mediated by antibodies against the glomerular basement membrane (Goodpasture disease); glomerulonephritis mediated by immunocomplexes (systemic lupus erythematosus, post-streptococcal, Henoch-Schonlein purpura) and Pauci-immune glomerulonephritis (ANCA-positive vasculitis with specificity for proteinase 3 - PR3 or myeloperoxidase - MPO). The latter compromises small vessels, generating more kidney damage in microscopic polyangiitis (MPA) and greater lung involvement in granulomatosis with polyangiitis (GPA). However, neither the organic compromise nor the positivity of the ANCAS can provide diagnostic certainty, considering that they can be positive in other pathologies such as SLE, endocarditis, inflammatory bowel disease, among others, and even in healthy populations. We present a case of a patient with RPGN and positive P-ANCA and renal biopsy, showing crescentic glomerulonephritis with a "full-house" pattern in indirect immunofluorescence and negative SLE autoimmune profile, which is an unusual association.

## Case description

**Figure 1:**
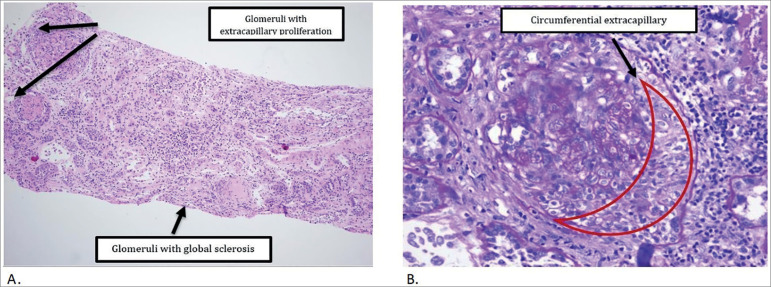
(A) Crescent glomerulonephritis with extracapillary cell proliferation and fibrocellular proliferation in approximately 66% of glomeruli, mediated by P-ANCAS. Glomerulonephritis mediated by immune complexes of superimposed membranoproliferative pattern. (B) Glomerulus with higher magnification (40X).

A 27 year old woman without an important clinical history, presented at the emergency room reporting 15 days of edema in the lower limbs and oliguria evolution. She referred weight gain in the last 15 days of approximately 5 kilograms. Upon physical examination, she presented with blood pressure in the range of hypertensive crisis, bipalpebral edema, jugular engorgement, basal crackles in both lung bases, and stage II edema with fovea in all four limbs. The admission test showed metabolic acidosis with elevated anion gap, blood count with leukocytosis, neutrophilia, normocytic normochromic anemia without transfusion criteria, electrolytes with severe hyperkalemia, KDIGO 3 acute kidney injury (Table 1), and electrocardiographic changes of hyperkalemia. In her initial approach as a hypertensive emergency with a compromised kidney versus a nephritic syndrome, medical management was started and, due to refractoriness, it was decided to perform hemodialysis in the nephrology service. Further studies with renal ultrasound showed chronic parenchymal process with signs of exacerbation, the patient also had increased phosphorus calcium profile with increased PTH, autoimmune profile with only one finding of positive P-ANCAS (1/160), and negative infectious profile (Table 2). The clinical evolution had persistent signs of overload and progressive deterioration of kidney function, and glomerulonephritis was considered rapidly progressive. Management began with pulses of intravenous corticosteroids for 3 days, plasmapheresis (7 sessions), and continuity of renal replacement therapy. A renal biopsy showed crescentic glomerulonephritis with P ANCAS-mediated extracapillary proliferation and glomerulonephritis mediated by immune complexes with superimposed membranoproliferative pattern, which is compatible with the full-house disease (Figure 1 and Table 3). Due to a satisfactory clinical evolution after the first dose of cyclophosphamide, she was discharged for continuation of immunosuppressive treatment and renal replacement therapy with hemodialysis.

**Table 1:** Initial tests results

	Reference value	Entry	1	2	3	Re-entry
**Hematological study**						
Hemoglobin (g/dL)	14.0-18.0	8.2	7.9	8.1	8.5	6.4
Hematocrit (%)	40.0-54.0	26.1	24.7	25.7	28.8	19.9
VCM (fl)	80.0-94.0	85.0	88.6	83.0	90.3	90.5
Leukocytes (cels/µL	4.500-11.500	13.900	12.850	8.544	7.541	28.490
Neutrophils (%)	50-70	88	77	69.1	67.8	86.2%
Platelets (mcL)	1500.000-450.000	198.000	145.000	155.000	201.000	267.000
**Renal function**						
Creatinine (mg/dL)	0.7-1.3	4.5	10.2	14.5	21.04	6.1
Blood urea nitrogen (mg/dL)	7-21	53	87.8	98.6	124.4	79
**Electrolytes**						
Sodium (mEq/L)	135-145	136	133	137	135	140
Potassium (mEq/L)	3.5-4.5	6.9	5.6	5.5	4.9	5.4
Chlorine (mEq/L)	96-106	103	101	99	101	98
**Proteins**						
Albumin (g/dL)	3.5-5	1.9				
Total proteins (g/dL)	6.4-8.3	5				
**Anion Gap (mEq/L)**	8-12	26.5				
Correct with albumin		28.4				
**Arterial blood gases**	pH: 7.32, PO2: 70.4, PCO2: 25.5, HCO3: 13.4, BE: -12.8, Lactate: 1.8 SATO2: 94% FIO2: 0.28					
**Uroanalysis**	Yellow, cloudy, urine density: 1010, pH: 6.0, Proteins: 500 mg/dL, Glucose: 50, Hemoglobinuria: 250 µL, Leucocytes 230 µL, Red blood cells: 135 µL, Bacteria: +					

**Table 2:** Extra test results

Autoimmune profile	
Anti MBG	Negative
ANAS	Negative
Anti-dsDNA	Negative
Anti-SSA RO:	Normal
Anti-SSB LA:	Normal
Anti-smith	Normal
ENAS	Normal
P ANCA	Positive
Complement	Normal
**Infectious**	
HIV	Nonreactive
Anti-HCV	Negative
HBsAg	Nonreactive
**Others**	
Peripheral blood smear	Normal
Reticulocytes	Normal
LDH	Normal
PTH	Normal
Match	Normal
Calcium	Normal

Anti MBG: anti-glomerular basement membrane antibodies; ANAS: antinuclear antibodies, Anti-SSA RO: antinuclear anti RO / SSA antibodies; Anti-SSB-LA: anti RO / SSB antinuclear antibodies, Anti-Smith: anti-Smith antinuclear antibodies, ENAS: removable antinuclear antibodies, P-ANCA: perinuclear neutrophil cytoplasmic antibodies; HIV: human immunodeficiency virus; Anti-HCV: anti hepatitis C antibodies; HbsAg: hepatitis B virus surface antigen; LDH: lactate dehydrogenase, PTH: parathyroid hormone.

**Table 3:** Results indicating "Full House" nephropathy. Deposits of all immunoglobulins were found, as well as C3 and C1q. Images were discarded

Antibody	Intensity and location
**Albumin**	-
**IgG**	+ mesangium
**IgA**	Mesangium traces
**IgM**	+++ mesangium
**C3**	+++ mesangium and capillary basement membrane
**C1q**	++ ++ mesangium and capillary basement membrane

**Figure 2:**
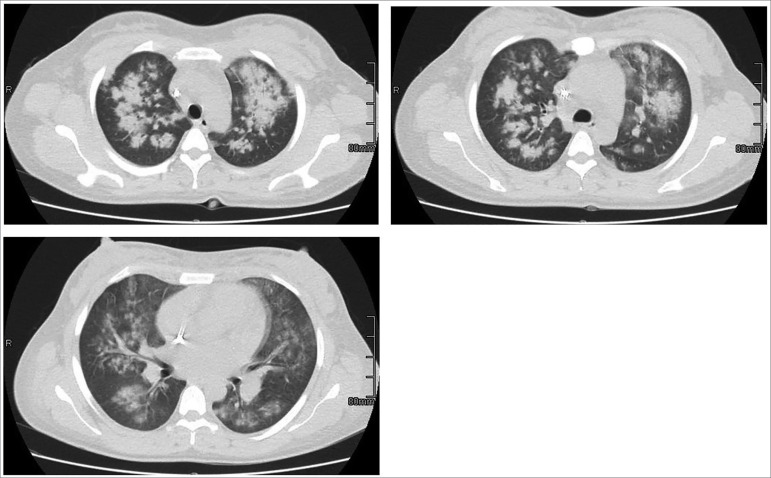
Axial cut in the the pulmonary window of high-resolution chest computed tomography. It is note extensive central alveolar occupation opacities located in both lung fields, with bilateral basal ground-glass areas in relation to multi-lobular consolidation compromise.

Five days after leaving the hospital, the patient was readmitted with a cough with purulent expectoration associated with fever, asthenia, and adynamia. The physical examination showed crackles in both lung bases and stage I edema in lower limbs. The patient underwent progressive deterioration of the respiratory pattern, anemization with transfusion requirement (Table 1), ground-glass imaging findings, and multi-lobular infiltrates (Figure 2). Antibiotic coverage (Cefepime) began and a decision was made to perform bronchial brushing bronchoscopy with macroscopic alveolar hemorrhage findings. The patient presented progressive deterioration of the ventilatory pattern, with the requirement of orotracheal intubation and transfer to intensive care unit. After presenting torpid clinical evolution, the patient died.

Microbiological screening studies (blood cultures, urine culture, bronchoalveolar lavage culture) were negative. The family did not authorize a necropsy.

## Discussion

RPGN is considered a medical emergency. It is a clinical syndrome characterized by proliferative extracapillary necrotizing crescent glomerulonephritis and rapid loss of kidney function, usually in days to months.^1^ Clinically, deterioration of renal function is observed (without current consensus on increased creatinine levels), glomerular inflammation (hematuria), proteinuria, anemia, oliguria or anuria, with or without hypertension and edema.^2^ Based on the immunofluorescence findings, the conditions is classified into 3 types: Type 1 refers to anti-glomerular basement membrane or Goodpasture syndrome with linear deposits, Type 2 is mediated by immunocomplexes with granular pattern, and Type 3 is Pauci-immune (ANCA-associated vasculitis)^3^. Its histological presentation depends on age; about 80% of crescentic pauci-immune GN are observed in patients >60 years, and the vast majority of young patients are mediated by immunocomplexes. Patient mortality has decreased due to new immunosuppressive therapies, going from 90% in the first year to around 20%^4^. Among the prognostic factors of the disease, age, cause, glomerular crescent compromise >80%, arterial sclerosis level, GFR <15 mL/min, and treatment are described.^5^^-^ 10 Data about the condition in the Colombian population are scarce, with unknown incidence. A series of 14 cases was published, with mean age at presentation of 44 years and ANCA-associated vasculitis (AAV) being the main etiology followed by immunocomplexes due to systemic Lupus erythematosus.^11^

Pauci-immune vasculitis is among the main causes of RPGN. The pathology leads to compromised small vessels, with characteristic up to 90% positivity for antibodies against neutrophil cytoplasm (ANCA) with specificity for PR3 or MPO^12^. An incidence of around 13 to 20 cases per million persons per year has been reported, with a prevalence of 46 to 184 cases/million individuals worldwide, a slightly preference for men, with a peak incidence between 60-70 years of age, higher in white and Asian races, and with mortality of up to 29% in 10 years^13^^,^ 14. Its classic classification is: microscopic polyangiitis (MPA), granulomatosis with polyangiitis (GPA), and granulomatosis with eosinophilic polyangiitis or Churg-Strauss syndrome (EGPA)^12^. Renal compromise in the MPA occurs in 90% of the cases, in contrast to a greater pulmonary compromise in 90% of the GPA.^15^ However, this classification is difficult due to the overlap between the syndromes, therefore the current trend is the classification according to the PR3 or MPO positivity^14^.

The serum positivity of the ANCAs does not give certainty of the diagnosis of pauci-immune vasculitis, since they are found in other conditions such as systemic lupus erythematosus (SLE), endocarditis, inflammatory bowel disease, primary sclerosing cholangitis, cystic fibrosis, and even in low percentages in healthy population^14^^,^ 16 ^-^ 18. On the other hand, its negativity does not exclude the disease, since it can be negative in around 10% of cases^14^. Antibody detection can be done by immunofluorescence (ANCA-P or ANCA-C) or by enzyme-linked immunoassay (Anti-MPO or Anti-PR3), the current recommendation being the last one due to reduced false positives.^19^^,^ 20 Anti-MPO has been observed to be more frequently associated with MPA (60%) and EGPA (up to 50%), unlike GPA with positive anti-PR3 around in 70% of cases^21^.

The case presented is about an RPGN in a young patient with no significant medical history, nor a previous significant clinical history of autoimmune conditions, presenting dialytic urgency with negative immunological studies, except for positive ANCA-P / MPO. The biopsy report described a crescentic glomerulonephritis with extracapillary cellular to fibrocellular proliferation in 66% of glomeruli mediated by P-ANCAS, with an overlapping immuno-complex glomerulonephritis and a full-house immunofluorescence pattern. Electron microscopy study was not performed because it was not available at the institution.

Due to the low prevalence of the RPGN etiologies and the age and sex of the patient, the main suspicion was SLE, but the immunological study results were negative for ANAS. Multiple case reports of seronegative lupus nephritis have been described, classified according to ACR criteria^22^ or SLICC^23^. However, through the new classification criteria for SLE by the EULEAR / ACR, the presence of ANAs >1:80 is strictly established as the entry criterion^24^. Since autoimmune diseases are epiphenomena in their serological behavior, patients present negative ANAs serologies at the beginning of the disease and after months or years become positive^25^. SLE and AAV are autoimmune diseases that can share clinical characteristics such as arthritis, skin lesions, and kidney involvement. In many cases, the two diseases can be distinguished by clinical characteristics, antibody profile, and kidney disease, but some patients may have mixed patterns including classification criteria for both SLE and AAV, called SLE/AAV overlap syndrome^26^^,^ 27.

Additional findings of the case from renal biopsy indicated a presentation more characteristic of SLE than AAV. The "full-house" immunofluorescence pattern is characterized by the presence of IgG, IgM, and IgA, and C3 and C1q deposits, which occurs mostly in lupus nephritis (71%), with a sensitivity of 71%, specificity of 90%, positive predictive value of 79%, and negative predictive value of 85%^28^. The pattern has been described to a lesser extent in other pathologies such as primary membranous glomerulopathy, IgA nephropathy, C1q nephropathy, infectious glomerulonephritis (endocarditis, hepatitis C, HIV), cryoglobulinemia, and ANCAs vasculitis^25^^,^ 28. The association of ANCAs vasculitis and full-house nephropathy has been described in a few studies in a very low proportion of up to 3%^28^.

Within the management of AAV in the context of RPGN, its immunosuppressive pillar is based depending on the severity of the disease, types of antibodies, and whether it is a relapse or debut. Generally, however, the recommendation is for the use of steroids at high doses and induction therapy with either cyclophosphamide or rituximab (with no superiority of one over the other), but the latter is recommended in conditions such as relapses, refractoriness, anti-PR3 positive or cyclophosphamide contraindication. On the other hand, the association with plasmapheresis is indicated in patients with creatinine >5.7 mg/dL, debut with dialytic urgency, or alveolar hemorrhage. Relapse-associated factors are young onset, anti-PR3 positivity, pulmonary involvement, adherence to medical treatment, and carrier of Staphylococcus aureus^14^^,^ 29. There are no studies about the management of patients with pauci-immune vasculitis and nephropathy with a full-house pattern.

In conclusion, we presented a case of ANCA-associated vasculitis with full-house kidney disease, an unusual condition with up to 3% presentation and few reports in the literature, highlighting the importance of its reporting and contribution to the literature.
